# Impaired Prefrontal Hemodynamic Maturation in Autism and Unaffected Siblings

**DOI:** 10.1371/journal.pone.0006881

**Published:** 2009-09-03

**Authors:** Yuki Kawakubo, Hitoshi Kuwabara, Kei-ichiro Watanabe, Michiko Minowa, Toshikazu Someya, Iwao Minowa, Toshiaki Kono, Hisami Nishida, Toshiro Sugiyama, Nobumasa Kato, Kiyoto Kasai

**Affiliations:** 1 Department of Neuropsychiatry, Graduate School of Medicine, University of Tokyo, Tokyo, Japan; 2 Department of Child Psychiatry, Graduate School of Medicine, University of Tokyo, Tokyo, Japan; 3 Tokyo Metropolitan Child Guidance Center, Tokyo, Japan; 4 Mie Prefectural Asunaro Hospital for Children and Adolescent Psychiatry, Tsu, Japan; 5 Aichi Children's Health and Medical Center, Obu, Japan; 6 Department of Psychiatry, Karasuyama Hospital, Showa University School of Medicine, Tokyo, Japan; Chiba University Center for Forensic Mental Health, Japan

## Abstract

**Background:**

Dysfunctions of the prefrontal cortex have been previously reported in individuals with autism spectrum disorders (ASD). Previous studies reported that first-degree relatives of individuals with ASD show atypical brain activity during tasks associated with social function. However, developmental changes in prefrontal dysfunction in ASD and genetic influences on the phenomena remain unclear. In the present study, we investigated the change in hemoglobin concentration in the prefrontal cortex as measured with near-infrared spectroscopy, in children and adults with ASD during the letter fluency test. Moreover, to clarify the genetic influences on developmental changes in the prefrontal dysfunction in ASD, unaffected siblings of the ASD participants were also assessed.

**Methodology/Principal Findings:**

Study participants included 27 individuals with high-functioning ASD, age- and IQ-matched 24 healthy non-affected siblings, and 27 unrelated healthy controls aged 5 to 39 years. The relative concentration of hemoglobin ([Hb]) in the prefrontal cortex was measured during the letter fluency task. For children, neither the [oxy-Hb] change during the task nor task performances differed significantly among three groups. For adults, the [oxy-Hb] increases during the task were significantly smaller in the bilateral prefrontal cortex in ASD than those in control subjects, although task performances were similar. In the adult siblings the [oxy-Hb] change was intermediate between those in controls and ASDs.

**Conclusion/Significance:**

Although indirectly due to a cross-sectional design, the results of this study indicate altered age-related change of prefrontal activity during executive processing in ASD. This is a first near-infrared spectroscopy study that implies alteration in the age-related changes of prefrontal activity in ASD and genetic influences on the phenomena.

## Introduction

Autism is diagnosed on the basis of a triad of behavioral features: impairment of reciprocal social interaction, communication and imagination, and the presence of repetitive and ritualistic behavior. Monozygotic twins are much more highly concordant for autism as compared to dizygotic twins [1], and the sibling risk rate for autism is much larger than the risk in the general population [2]. Relatives of individuals with autism exhibit higher than normal rates of autism-related impairments including language, communication and social behavior, referred to as the broader autism phenotype (BAP) [3], [4]. These findings provide evidence that there is a heritable component to autism. A recent twin cohort study suggests that the elements of the triad of behavioral features of autism are largely independent of each other in terms of genetic background [5]–[8], thus, it is important to clarify the brain substrates for fundamental cognitive functions and identify biological or behavioral markers of genes contributing specifically to each of the three behavioral features of autism.

Dysfunctions of prefrontal cortex have been previously reported in individuals with autism spectrum disorders (ASD). Most neuropsychological studies have shown impairments of executive function, including planning, flexibility and working memory [8]. In functional magnetic resonance imaging (fMRI) studies, individuals with ASD have abnormalities of hemodynamic responses in the prefrontal cortex associated with spatial working memory [9]–[11], motor inhibition [12] and visuomotor control [13]. Our research group has recently reported that prefrontal activation assessed with 24-channel near-infrared spectroscopy (NIRS) [14] during the letter fluency task was reduced in adults with ASD as compared with healthy adults. These prefrontal activation studies present only findings of adults and adolescents with ASD, but not those of children with ASD. Moreover, previous studies did not evaluate data from both children-adolescents and adults using the same activation task. Thus, developmental changes in the prefrontal dysfunction in ASD are unclear.

In studies on first-degree relatives with ASD, various *neuropsychological* evaluations have been conducted to uncover a possible neurocognitive endophenotype of autism. Similar to individuals with ASD, their parents showed poor performance on Eyes test [15], which was used to assess the ability of estimating the mental state of others. Moreover, parents showed superior language abilities [16], weak central coherence [17], [18] and poor planning and attention flexibility [19]. Relatives included parents and child and adult siblings showed poor executive function [20]. Although there were no studies for only adult sibling, both child and adult siblings showed superior language abilities [16]. For child siblings, some studies have reported poor performance on Eyes test [15], poor planning, flexibility [21], [22] and verbal fluency [22]. Other studies, however, have reported the intact central coherence [17], planning [23] and language abilities [24], [23]. Taken together, previous literature on neurocognitive dysfunction in relatives of individuals with ASD has been divergent, which may partly depend on whether the study participants were children or adults. These inconsistencies might be due to differences in neurocognitive function at different developmental stages. Specifically, group differences might not be detected in childhood because the prefrontal cortex is not mature until adolescence even in typically developing children. Thus, in order to clarify prefrontal function in siblings of individuals with ASD, further research that uses the same task for both child and adult siblings is necessary.

To date, little empirical data have been available regarding the brain functional imaging of first-degree relatives of individuals with ASD. In fMRI studies, child siblings of individuals with ASD showed decreased gaze-fixation along with diminished activation in the fusiform gyrus compared with the control group [25], and parents of individuals with ASD showed the hyper-masculinization of the brain during both visual search and emotion recognition [26]. An event-related potential (ERP) study reported atypical face processing in parents of individuals with ASD [27]. To our knowledge, however, no previous studies have examined prefrontal cortex activation associated with executive function in first-degree relatives of individuals with ASD.

NIRS is one of the most promising functional neuroimaging tools to allow comparative evaluation of cortical hemodynamic response between children and adults with ASD using a common apparatus. NIRS can noninvasively measure the relative concentrations of oxy-hemoglobin ([oxy-Hb]) and deoxy-hemoglobin ([deoxy-Hb]), which are assumed to reflect regional cerebral blood volume (rCBV), in a natural experimental setting. While fMRI and PET have an excellent spatial resolution, they are limited in that they require large apparatuses that prevent their use in bedside settings for diagnostic and treatment purposes. In contrast, NIRS is a neuroimaging modality that, for the following reasons is especially suitable for assessing the prefrontal cortex of children [28] and psychiatric disorders [29]–[32]. First, NIRS can be applied to experiments that might cause some motion of the subjects such as vocalization. Second, the subject can be examined in a natural sitting position, without any surrounding distraction. Third, the cost is much lower than other neuroimaging modalities and the set-up is very easy. Fourth, the high temporal resolution of NIRS is useful in characterizing the time course of prefrontal activity [29]–[31].

By simultaneous measurements with other methodologies, it has been shown that the [oxy-Hb] change measured by NIRS correlates with the rCBF change in 15H2O PET [33] and the blood oxygenation level-dependent [34] signal in fMRI [35]. In other fMRI studies [34], [36], [37], in which the [oxy-Hb] change was not analyzed, the [deoxy-Hb] change in NIRS has been correlated with the BOLD signal.

Moreover, previous lesion study provided that frontal cortex is needed to perform the letter fluency test [38], [39]. Previous NIRS studies showed that the verbal fluency test is a valid cognitive activation task to evaluate [Hb] change in prefrontal cortex using NIRS [14], [30], [31], [33], [40]. In NIRS studies recording the [Hb] changes during several tasks for the same subject group, the smaller-than-normal [oxy-Hb] change during the frontal function task, such as the letter fluency test and random number generation task, in schizophrenia was task specific, i.e., this was not evident during other tasks (sequential finger-to-thumb task[41] and finger tapping task[30]). These findings suggest that the [oxy-Hb] change reflected the neural activation but not general and nonspecific factors, such as impaired vascular responsiveness irrespective of neural activation and optical pathlength.

In the present study, we investigated the change in hemoglobin concentration in the prefrontal cortex as measured with NIRS, in children and adults with ASD during the letter fluency test. Moreover, to clarify the genetic influences on developmental changes in the prefrontal dysfunction in ASD, unaffected siblings of the ASD participants were also assessed.

## Methods

### Participants

Participants were 27 individuals with high-functioning (i.e. IQ≥85) ASD (21 male and 6 female) who met criteria for autistic disorder, Asperger's disorder or pervasive developmental disorder not otherwise specified (PDD-NOS) in DSM-IV, 24 healthy non-affected siblings with ASD (11 male and 13 female), and 27 unrelated healthy controls with no family history of ASD (21 male and 8 female). Each group was divided into two subgroups by age: a child group and an adult group (Table 1). We need to replicate Kuwabara et al [14] with a different NIRS instrument and study participants. Therefore, we separated at 18 years old in order to set the age range for adults group to the same as adopted in Kuwabara et al. [14]. In both groups, mean age and IQ were matched among individuals with ASD, non-affected siblings and unrelated healthy controls. For sex ratio, however, there was a significant difference in the child group (ASD: 12 male, 2 female; non-affected siblings: 4 male, 8 female; unrelated healthy controls: 12 male, 2 female). However, as described in the results section, sex distribution did not significantly influence the conclusions of the study. Individuals with ASD and non-affected siblings were recruited from outpatients of the Department of Neuropsychiatry and the Department of Child Psychiatry, University of Tokyo Hospital, Japan, the Mie Prefectural Asunaro Hospital for Children and Adolescent Psychiatry, Japan, and the Aichi Children's Health and Medical Center, Japan and from participants of public symposiums about ASD held at the University of Tokyo. Individuals with ASD and non-affected siblings were assessed for symptoms of ASD according to the Childhood Autism Rating Scale-Tokyo Version (CARS-TV) [42] by trained child psychiatrists (H.K., M.M. and K.W.). Non-affected siblings were evaluated for the presence of BAP [3], and no siblings were considered as having BAP. Ten out of 14 children and 10 out of 13 adults with ASD were on psychotropic medication at the time of examination (Table 2). Healthy controls were mainly recruited from college students, hospital staff and their acquaintances and children. The exclusion criteria for all groups were neurological illness, traumatic brain injury with any known cognitive consequences or loss of consciousness for more than 5 minutes, a history of electroconvulsive therapy, and alcohol/substance abuse or addiction. An additional exclusion criterion for the healthy control group was a history of psychiatric disease in themselves or a family history of axis I disorder in their first-degree relatives. IQs were evaluated with the WISC-III or WAIS-R. All participants were right-handed as based on the Edinburgh Inventory [43], except 1 ASD child and 1 sibling child (exclusion of the subject did not change statistical conclusions).

**Table 1:** Characteristics of Participants.

**Child group**		**Diagnosis**		
	**ASD**	**Sibling**	**Control**	
	**n = 14**	**n = 12**	**n = 14**	
**m/f**	12/2	4/8	12/2	p<.01
**age**	12.7±3.4	11.1±3.0	10.6(2.8	p = .25
**(range)**	(6.6(17.3)	(5.9(15.8)	(5.8(16.6)	
**IQa**	95.2(8.4	103.5(12.9	102.1(6.5	p = .065
**(range)**	(85(108)	(85(121)	(90(114)	
**CARS**	28.7(5.3	15.4(0.8	----	p<.001
**(range)**	(22(40)	(15(17.5)	----	
**Adult group**		**Diagnosis**		
	**ASD**	**Sibling**	**Control**	
	**n = 13**	**n = 12**	**n = 13**	
**m/f**	9/4	7/5	9/4	p = .81
**age**	26.7±6.1	24.3±4.3	25.8±5.1	p = .53
**(range)**	(18.3∼39.0)	(18.9∼35.4)	(21.4∼37.4)	
**IQ** b	104.7±11.5	105.4±8.7	111.3±12.6	p = .27
**(range)**	(86∼123)	(92∼117)	(92∼128)	
**CARS**	29.6±6.6	15.6±0.8	----	p<.001
**(range)**	(19∼40.5)	(15∼17.5)	----	

CARS, Childhood Autism Rating Scale; f, female; m, male;

a IQ was evaluated with the WISC-III, except for 1 ASD child estimated IQ by four subtests of the WAIS-R.

b For all ASDs and 4 siblings IQs were evaluated with the WAIS-R, for 8 siblings and all controls IQs were estimated by four subtests of the WAIS-R.

**Table 2:** The individual's data in ASD group.

	case	sex	age	handedness	subtype	CARS	medication (mg/day)	EIQ	VIQ	PIQ	FIQ	LFT
Child	1	f	6.6	R	Au	35.5	none	.	104	100	102	3
	2	m	7.6	R	Au	31	none	.	77	99	86	5
	3	m	8.9	R	Au	40	Risperidone 1, Sodium valproate 200	.	101	94	98	5
	4	m	9.3	R	As	32.5	Levomepromazine 10, Lithium 200, Carbamazepine 200	.	101	113	107	2
	5	m	10.6	R	Au	27	Risperidone 2	.	85	107	95	1
	6	f	13.0	R	PDD-NOS	23	none	.	120	93	108	6
	7	m	13.5	R	Au	31.5	Pimozide 3, Levomepromazine 5, Clonazepam 1	.	86	100	92	3
	8	m	13.5	R	As	23	Sulpiride 300, Estazoram 2	.	87	85	85	7
	9	m	14.0	R	PDD-NOS	22	none	.	104	94	99	6
	10	m	14.1	R	As	30.5	Haloperidol 9, Sodium valproate 600	.	87	87	86	2
	11	m	15.2	L	PDD-NOS	22.5	Risperidone 2, Zotepine 75, Lithium 200, Nitrazepam 5	.	81	99	88	7
	12	m	16.2	R	PDD-NOS	28.5	Risperidone4, Chlorpromazine 25, Levomepromazine 200, Carbamazepine 800,	.	84	92	86	4
							Fluboxamine 75, Nitrazepam 5, Pentobarbital 50, Phenobarbital 40					
	13	m	17.3	R	As	28	Haloperidol 4, Levomepromazine 150, Carbamazepine 1200	106	.	.	.	5
	14	m	17.6	R	As	27	Risperidone 2, Carbamazepine600, Sodium valproate400	.	96	94	95	8
Adult	1	f	18.3	R	PDD-NOS	25	none	.	108	91	101	4
	2	m	20.4	R	As	33.5	Carbamazepine 200, Paroxetine 20, Brotizolam 0.5	.	101	101	101	4
	3*	m	21.9	R	PDD-NOS	19	Methylphenidate	.	98	108	102	8
	4	m	22.1	R	As	26	Sodium valproate 200, Paroxetine 20, Ethyl loflazepate 1	.	103	90	98	8
	5	m	23.8	R	As	35	None	.	109	95	104	12
	6	m	24.0	R	Au	38	Risperidone 4, Quetiapine450, Chlorpromazine 25, Sodium valproate 600,	.	78	105	86	8
							Fluvoxamine 100, Bromazepam 4					
	7*	m	26.0	R	PDD-NOS	20.5	Risperidone, Fluboxamine, Etizolam	.	97	95	95	8
	8	m	26.0	R	As	40.5	Setiptiline 2, Triazolam 0.25, Ethyl loflazepate 1	.	100	130	113	7
	9	f	28.3	R	As	35	Fluvoxamine 75	.	74	113	90	5
	10	m	29.5	R	As	24.5	Sodium valproate 600, Fluboxamine 75	.	136	103	123	9
	11	f	31.1	R	As	30	None	.	121	103	114	10
	12	f	36.3	R	As	29.5	Pimozide 1, Clomipramine 20	.	124	116	122	10
	13	m	39.0	R	As	28	Sodium valproate 200, Fluvoxamine 100, Zolpidem 5	.	113	108	112	9

CARS, Childhood Autism Rating Scale; EIQ, estimated IQ; VIQ, verbal IQ; PIQ, performance IQ; FIQ, Full-scale IQ; LFT, the number of words generated during the letter fluency task; L, left; R, right; f, female; m, male; Au, autistic disorder; As, asperger disorder; PDD-NOS, pervasive developmental disorder not otherwise specified.

* The dosages for Case 3 and 7 were unavailable.

### Ethics

The ethical committees of the University of Tokyo Hospital and the Aichi Children's Health and Medical Center approved this study (receipt No. 630–5). The Mie Prefectural Asunaro Hospital for Children and Adolescent Psychiatry delegated the ethical review to the ethical committee of the University of Tokyo Hospital because they did not have an institutional review board. All adult participants gave written informed consent. All child participants gave informed assent and their parents gave written informed consent.

### Activation task

The activation task consisted of a 30 sec rest, a 30 sec letter fluency task and 30 sec rest. In the letter fluency task, participants were asked to generate as many words that began with a syllable /a/ as they could. The participants sat on a chair with their eyes open and hold their hands on their lap throughout the measurement. The auditory cues were presented at the start and end of the letter fluency task or rest and the syllable was also presented as the auditory cue at the start of the task. Hemoglobin concentration changes were measured during the activation task. The activation task was similar to that in previous studies [14], [30], but 3 changes were introduced to make the task suitable for children: (1) In the pre- and post-task participants were silent instead of repeating syllables; (2) The time period of the letter fluency task and post-task was shortened to 30 sec from 60 sec; (3) Only a single syllable was used in the letter fluency task. The number of words generated during the letter fluency task was determined as a measure of task performance.

### NIRS measurement

The relative concentration of oxyhemoglobin [oxy-Hb] and deoxyhemoglobin [deoxy-Hb] was measured using a 2-channel NIRS machine (NIRO200, Hamamatsu Photonics, Inc) at three wavelengths of near-infrared light (775, 810, 850 nm). The measurement principles are based on the modified Beer-Lambert law, which calculates the [oxy-Hb] and [deoxy-Hb] concentration changes from the light attenuation change at a given measured point. With a pathlength of 24 cm the concentration changes of [oxy-Hb] and [deoxy-Hb] are given in the unit micro mol. Each of the two probes consisted of an emitter and a detector separated by 4 cm. The two NIRS probes were placed on the subject's prefrontal regions and secured using double-sided adhesive tape such that the detectors were positioned at Fp1 and Fp2 with the emitters were positioned on 4 cm lateral side of detectors along the T3-T4 line according to the international 10/20 system. The machine measures [Hb] change approximately 2–3 cm beneath the scalp, i.e., the cortical surface area [33], [36]. NIRS measured oxygenation in approximately Brodmann's areas 10 [44]. The sampling time for the recording was 0.5 sec. Baseline correction was made by using the average [Hb] change during the first 30 s rest, and then the average [Hb] change during the 30 sec task period was calculated in each hemisphere.

### Statistical methods

The child and adult groups were analyzed separately. For the mean [Hb] change during the 30 sec task period, ANOVA was performed with Diagnosis (ASD, sibling, control) as the between-subjects factor and Hemisphere (left, right) as the within-subjects factor. A 1-way ANOVA was used to analyze task performance. When the sphericity assumption was violated, Greenhouse-Geisser correction was applied and the associated epsilon was reported. The tukey's honestly significant difference test was used for post hoc multiple comparisons.

We focused on the [oxy-Hb], since the [oxy-Hb] change is assumed to more directly reflect cognitive activation than the [deoxy-Hb] change as shown by a stronger correlation with blood-oxygenation level–dependent signal measured by fMRI [35]. Thus, the ANOVAs on [deoxy-Hb] data were also briefly reported. Moreover, we calculated Pearson's correlation between [oxy-Hb] change at each hemisphere and task performance, age and IQ separately for each diagnosis and for child/adult group. Additionally, Spearman's rank correlation coefficients were calculated for a relationship between the [oxy-Hb] change at each hemisphere and CARS scores for ASD, separately for child/adult groups.

## Results

### 1) Task performance

The mean number of words generated during the letter fluency task was: 4.14 (SD = 2.11) in control child group, 5.33 (SD = 2.31) in sibling child group, 4.57 (SD = 2.14) in ASD child group, 8.92 (SD = 2.90) in control adult group, 7.25 (SD = 3.14) in sibling adult group and 7.85 (SD = 2.38) in ASD adult group. Task performances were not significantly different among the three diagnoses, neither in child nor adult group, (Child: F(2,37) = .978, p = .39; Adult: F(2,35) = 1.15, p = .33).

### 2) [Hb]

#### 2-1 Child group (*Figure 1*)

The average [Hb] changes in both hemispheres were shown in Table 3. For the [oxy-Hb] change, neither the main effects nor the interaction were significant (Diagnosis: F(2,37) = 2.37, p = .11; Hemisphere: F(1,37) = .099, p = .76; interaction: F(2,37) = 1.98, p = .15). The average [oxy-Hb] change was not significantly different among the three diagnoses.

**Figure 1:**
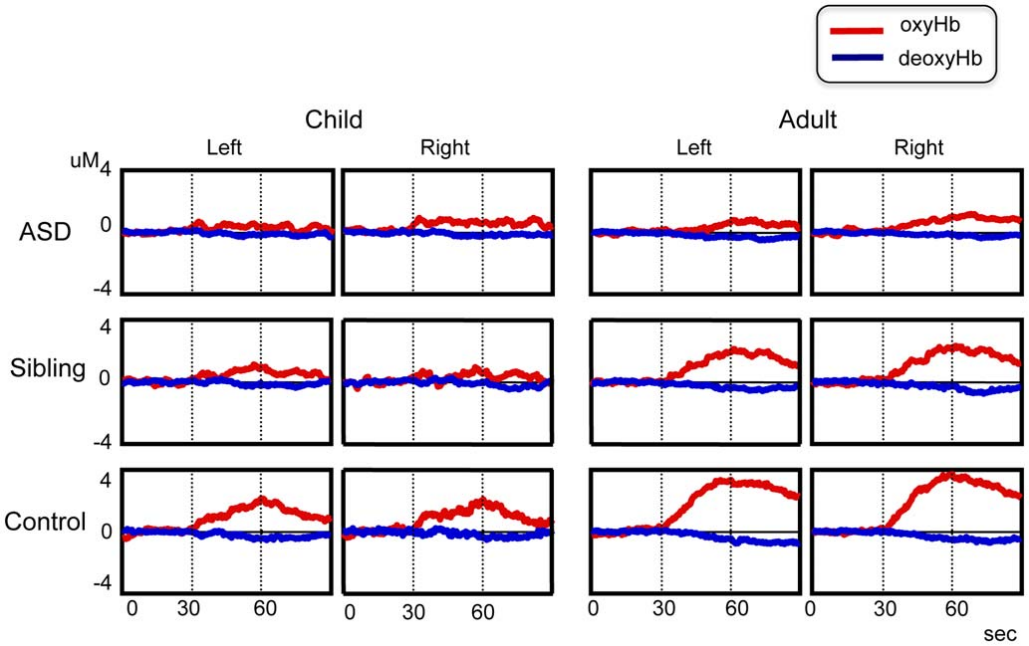
Grand average waveforms of hemoglobin concentration changes during the letter fluency task. Top: Autism spectrum disorder, Middle: Unaffected siblings, bottom: healthy control, right: adult group, left: child group. Line: red, oxyhemoglobin; blue, deoxyhemoglobin. The period of the activation task is between two dot-lines.

**Table 3:** Mean [Hb] (SD) in each group.

child group	oxyHb	deoxyHb
	R	L	R	L
ASD	0.65(0.89)	0.38(0.88)	−0.08(0.32)	−0.17(0.25)
Sibling	0.41(0.73)	0.59(0.75)	0.01(0.55)	−0.05(0.33)
Control	1.17(1.21)	1.16(1.12)	−0.10(0.34)	−0.22(0.32)

adult group	oxyHb	deoxyHb
	R	L	R	L
ASD	0.53(1.14)	0.21(1.17)	−0.12(0.25)	−0.20(0.28)
Sibling	1.29(1.41)	1.05(1.12)	−0.21(0.38)	−0.21(0.38)
Control	2.17(1.92)	2.05(2.04)	−0.23(0.55)	−0.20(0.55)

oxyHb, oxyhemoglobin; deoxyHb, deoxyhemoglobin; L, left; R, right.

Also for the [deoxy-Hb] change, there was no significant difference (Diagnosis: F(2,37) = .645, p = .53; Hemisphere: F(1,37) = 2.803, p = .10; interaction: F(2,37) = .094, p = .91).

#### 2-2 Adult group (*Figure 1*)

Table 3 showed the average [Hb] changes in both hemispheres. For the [oxy-Hb] change, there was a significant main effect of Diagnosis (F(2, 35) = 4.48, p = .02) and Hemisphere (F(1,35) = 4.23, p = .05). The interaction was not significant (F(2,35) = .29, p = .75). The average [oxy-Hb] in right hemisphere was significantly larger than that in left hemisphere. The average [oxy-Hb] in ASDs was significantly smaller than that in controls (p = .014).

However, the [deoxy-Hb] change was not significantly different (Diagnosis: F(2,35) = .094, p = .91; Hemisphere: F(1,35) = .07, p = .79; interaction: F(2,35) = .272, p = .76).

### 3) Correlational analysis (*Figure 2*)

Mean [oxy-Hb] changes were not significantly correlated with task performance and IQ in any of the groups. For the correlation between the [oxy-Hb] change and age, the [oxy-Hb] change at left hemisphere was positively correlated with age only in the child sibling group (r = 0.58, p = 0.046), although this correlation did not remain significant after Bonferroni correction. There was no significant correlation between the [oxy-Hb] change and CARS scores in the ASD group.

**Figure 2:**
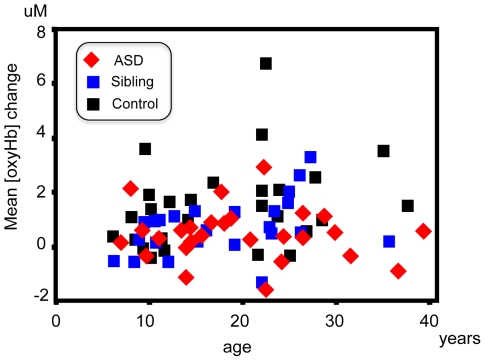
The scatter plot of age and the mean [oxy-Hb] change between hemispheres. Red: autism spectrum disorder, blue: unaffected siblings, black: healthy control.

### 4) Effects of sex ratio and medication

Mann-Whitney U test showed that [oxy-Hb] change was not significantly different between sexes for any group (z<0.91; p>0.36). Moreover, medicated and unmedicated subjects with ASD did not significantly differ in left or right PFC [oxy-Hb] change for either the child (Mann-Whitney U test: p's [left/right] = 0.48/0.12) or the adult group (p's [left/right] = 0.87/0.31) (Table 2).

## Discussion

To our knowledge, this is the first report of a contrast of prefrontal hemodynamic data between ASD and their unaffected siblings. First, in the children the [oxy-Hb] change during the task was small and there were no significant differences in the [oxy-Hb] change among the three diagnostic groups. Second, in the adults the ASDs showed smaller [oxy-Hb] change than the controls, and the [oxy-Hb] change in the adult siblings was intermediate between those in controls and ASD. Regarding the development of the prefrontal cortex, previous studies on postmortem brain, structural MRI, and PET indicate that maturation appears to continue into late adolescence [45]–[49]. Furthermore, an fMRI study investigated prefrontal activation during the declarative memory task for 8–24 years old [50] and an NIRS study using the stroop task in 7–13 year olds [28] also reported that prefrontal activation increases with age. Taken together, although indirectly due to a cross-sectional design, the results of this study indicate altered age-related change of prefrontal activity during executive processing in ASD. Furthermore, the results of unaffected siblings indicate that some genetic factors may influence this developmental deficit of prefrontal cortex in ASD.

The present study suggests that recruitment of prefrontal cortex (approximately BA10; frontopolar regions, also known as anterior prefrontal cortex in this study) during executive tasks increases with maturation in healthy individuals, but that this is not fully accomplished in individuals with ASD. An fMRI study using the verbal fluency task found that activation of the ventrolateral prefrontal cortex (BA44/45) is larger in children than in adults [51]. In contrast, the frontopolar regions, (corresponding approximately to the location measured by NIRS in the present study), have a higher-order integrative prefrontal function [52] and comparative studies of humans and apes [53] suggest that they have enlarged and become specialized during hominid evolution. The frontopolar regions might coordinate ventrolateral and dorsolateral functions in order to achieve task goals or maximize task performance [54]–[56], and might evaluate internally generated information [57]. Thus, although speculative, the cortical area recruited by the verbal fluency task might shift from the dorso-ventrolateral to the anterior polar region with age. However, this interpretation with respect to activation moving between different prefrontal areas during maturation should be investigated in future studies using a instrument with a wider coverage of prefrontal and temporal area.

In the unaffected adult siblings, [oxy-Hb] change is intermediate between the controls and the ASD group. Previous studies reported that first-degree relatives of individuals with ASD show atypical brain activity during tasks associated with social function, such as emotion recognition [26], face processing [27] and gaze processing [25]. Our observations suggest that developmental deficit of the prefrontal cortex associated with impaired executive function represents a genetic trait for ASD.

The adult healthy control group showed prefrontal activation during the verbal fluency task, which is in accordance with previous studies using fMRI and PET [58], [59] and NIRS [14], [30], [31], [33], [40]. Furthermore, the present results of the adults with ASD replicated the findings of reduced prefrontal activation in a previous NIRS study using a different NIRS apparatus (ETG-100, Hitachi Medical Co., a 24-channel machine) in an independent adult sample [14]. Thus, although there are some technical/methodological differences between the different NIRS apparatuses such as the wavelength of near-infrared light, [Hb] value and sampling time, they did not significantly affect the results of comparison between patients and controls. However, for a direct comparison of the data obtained from the difference apparatuses, a rigid verification is needed by a simultaneous measurement using different NIRS apparatuses and a repeated measurement for an identical subject using different NIRS apparatuses. We used the resting state as the baseline to facilitate applicability of the task to child participants. The smaller [oxy-Hb] change in adults with ASD may reflect the difference in the baseline due to hyperperfusion in the ASD group. This is because NIRS does not measure absolute Hb concentration, but rather change relative to Hb concentration during the baseline task. Thus, if adults with ASD are hyperperfused during the baseline, activation during the task will be measured as smaller than equivalent activation in the control group. PET studies, however, have reported hypoperfusion during the resting state in the frontal areas of children and adults with ASD [60], [61]. Furthermore, an fMRI study has shown that prefrontal activation in the resting state decreases in adults with ASD [62]. Thus, the smaller [oxy-Hb] change in ASD might not be due to hemodynamic state during rest but due to reduced prefrontal activation during the task.

ASD adults did not show significant prefrontal activation during the task in spite of preserved task performance. These results suggest that ASD is associated with developmental deficit of frontopolar recruitment associated with verbal fluency. However, our study using the 2-channel NIRS cannot conclude the specificity to prefrontal dysfunction and cannot exclude the possibility that other regions of the prefrontal cortex and superior temporal regions normally recruited in adults during verbal fluency task [40], [58] were hyperactivated in ASD. Previous fMRI and PET studies have reported that the prefrontal cortex in adults with ASD is under-activated while other regions are activated normally or over-activated [11], [63]. Thus, further research using machines with wider coverage of cortical areas will be necessary to test this possibility.

Other methodological considerations should be commented upon. First, in the child group sex was not matched among the three diagnostic groups although sex distribution may not significantly influence the conclusions of the study. Second, most of the participants with ASD were on various psychotropic drugs. Although there were no effects of medication in this study, further studies for the non-medicated patients are needed.

In conclusion, to our knowledge this is the first study that investigated prefrontal cortical function in children and adult individuals with ASD and unaffected siblings. Adults with ASD and, to a lesser extent, unaffected siblings show decreased prefrontal activation. Further studies are needed to determine whether it reflects developmental deficit of the prefrontal cortex that may be related to the genetic risk associated with ASD.
